# Overlapping Residual Herbicides for Control of Photosystem (PS) II- and 4-Hydroxyphenylpyruvate Dioxygenase (HPPD)-Inhibitor-Resistant Palmer amaranth (*Amaranthus palmeri* S. Watson) in Glyphosate-Resistant Maize

**DOI:** 10.3389/fpls.2017.02231

**Published:** 2018-01-09

**Authors:** Parminder S. Chahal, Zahoor A. Ganie, Amit J. Jhala

**Affiliations:** Department of Agronomy and Horticulture, University of Nebraska-Lincoln, Lincoln, NE, United States

**Keywords:** net return, PRE followed by POST, residual herbicides, resistance management, weed management

## Abstract

A Palmer amaranth (*Amaranthus palmeri* S. Watson) biotype has evolved resistance to photosystem (PS) II- (atrazine) and 4-hydroxyphenylpyruvate dioxygenase (HPPD)-inhibiting herbicides (mesotrione, tembotrione, and topramezone) in maize seed production field in Nebraska, USA. The objectives of this study were to determine the effect of soil residual pre-emergence (PRE) herbicides followed by (fb) tank-mixture of residual and foliar active post-emergence (POST) herbicides on PS-II- and HPPD-inhibitor-resistant Palmer amaranth control, maize yield, and net economic returns. Field experiments were conducted in a grower's field infested with PS II- and HPPD-inhibitor-resistant Palmer amaranth near Shickley in Fillmore County, Nebraska, USA in 2015 and 2016. The contrast analysis suggested that saflufenacil plus dimethenamid-P or pyroxasulfone plus saflufenacil applied PRE provided 80–82% Palmer amaranth control compared to 65 and 39% control with saflufenacil and pyroxasulfone applied alone at 3 weeks after PRE (WAPRE), respectively. Among the PRE fb POST herbicide programs, 95–98% Palmer amaranth control was achieved with pyroxasulfone plus safluefenacil, or saflufenacil plus dimethenamid-P applied PRE, fb glyphosate plus topramezone plus dimethenamid-P plus atrazine, glyphosate plus diflufenzopyr plus dicamba plus pyroxasulfone, glyphosate plus diflufenzopyr plus pendimethalin, or glyphosate plus diflufenzopyr plus dicamba plus atrazine applied POST at 3 weeks after POST (WAPOST) through maize harvest. Based on contrast analysis, PRE fb POST programs provided 77–83% Palmer amaranth control at 3 WAPOST through maize harvest compared to 12–15% control with PRE-only and 66–84% control with POST-only programs. Similarly, PRE fb POST programs provided 99% biomass reduction at 6 WAPOST compared to PRE-only (28%) and POST-only (87%) programs. PRE fb POST programs provided higher maize yield (13,617 kg ha^−1^) and net return (US $1,724 ha^−1^) compared to the PRE-only (2,656 kg ha^−1^; US $285 ha^−1^) and POST-only (11,429 kg ha^−1^; US $1,539 ha^−1^) programs. The results indicated that effective control of multiple herbicide-resistant Palmer amaranth can be achieved with PRE fb POST programs that include herbicides with overlapping residual activity to maintain season-long control.

## Introduction

Palmer amaranth is a summer annual broadleaf weed species belonging to the family Amaranthaceae that has separate male and female plants (Sauer, 1957). Palmer amaranth is a prolific seed producer and if left uncontrolled, a single female plant can produce as many as 600,000 seeds (Keeley et al., 1987). Palmer amaranth has the highest specific leaf area (149–261 cm^2^ g^−1^), photosynthetic rate (80 μmol CO_2_ m^−2^ s^−1^), and growth rate (0.10–0.21 cm per growing degree day) out of all of the *Amaranthus* species (Horak and Loughin, 2000). Palmer amaranth can tolerate medium to mild water stress conditions using osmotic adjustment as a drought tolerance mechanism (Ehleringer, 1983). Furthermore, Palmer amaranth populations have been reported resistant to microtubule-, acetolactate synthase (ALS)-, photosystem (PS) II-, 5-enol-pyruvylshikimate-3-phosphate synthase (EPSPS)-, 4-hydroxyphenylpyruvate dioxygenase (HPPD)-, and protoporphyrinogen oxidase (PPO)-inhibiting herbicides in different states throughout the USA (Heap, 2017). Palmer amaranth biotypes with multiple resistance to two or more herbicide sites of action have also been confirmed (Sosnoskie et al., 2011; Nandula et al., 2012; Heap, 2017). Palmer amaranth's aggressive growth habits and prolific seed production along with its evolution of resistance to different herbicide sites of action has made it the most problematic crop weed in the USA (Horak and Loughin, 2000; Berger et al., 2015; Chahal et al., 2015, 2017; Kohrt and Sprague, 2017).

A PS II- (atrazine) and HPPD-inhibitor-resistant Palmer amaranth biotype has been reported in a continuous maize seed production field in south-central Nebraska, USA (Jhala et al., 2014). While rapid detoxification and increased HPPD gene expression was reported as the mechanism conferring resistance to HPPD-inhibitor in the Palmer amaranth biotype from Nebraska (Nakka et al., 2017), the mechanism of atrazine resistance in this biotype is unknown. PS II- (atrazine) and HPPD-inhibitor (mesotrione, tembotrione, or topramezone) are the most commonly used herbicides for weed control in maize due to their pre-emergence (PRE) and post-emergence (POST) activity, broad-spectrum weed control, and crop safety, particularly in sweet maize, seed maize, and maize for popcorn (Fleming et al., 1988; Swanton et al., 2007; Bollman et al., 2008). The evolution of Palmer amaranth resistant to PS II- and HPPD-inhibitor has reduced the number of herbicide options for Palmer amaranth control in maize in Nebraska, USA.

The management of herbicide-resistant (HR) Palmer amaranth requires PRE followed by (fb) POST herbicide programs with distinct sites of action, herbicide rotation, and rotation of HR crop traits (Jhala et al., 2014; Crow et al., 2016; Chahal et al., 2017). The majority of the maize fields in Nebraska are under glyphosate-resistant (GR) maize production systems using either single or sequential glyphosate applications for POST weed control (Jhala et al., 2014; Chahal et al., 2017). Studies conducted in Nebraska have reported that the PS II- and HPPD inhibitor-resistant Palmer amaranth biotype is sensitive to glyphosate applied at the labeled rate because glyphosate had not been used over the past 8 years while the field was kept under continuous maize seed production (unpublished data). Therefore, glyphosate can be considered as one of the herbicide options for management of PS II- and HPPD inhibitor-resistant Palmer amaranth in GR maize. In Nebraska, GR weed species, including common ragweed (*Ambrosia artemisiifolia L*.), common waterhemp (*Amaranthus rudis Sauer*), horseweed [*Conyza canadensis (L.) Cronq*.], giant ragweed (*Ambrosia trifida L*.), and kochia [*Kochia scoparia (L.) Schrad*.] have been reported (Sarangi et al., 2015; Chahal et al., 2017; Ganie and Jhala, 2017a; Heap, 2017). More recently, GR Palmer amaranth has also been confirmed in Nebraska (Chahal et al., 2017). In view of the widespread occurrence of six GR broadleaf weeds in Nebraska, tank-mixing glyphosate with other site of action herbicides and rotation of GR maize with other HR crop traits has become important to diversify the number of herbicide options for management of HR weeds such as Palmer amaranth (Ganie et al., 2017; Ganie and Jhala, 2017b).

Palmer amaranth has an extended period of emergence (March–October) in the midwestern and southern USA, making it difficult to control, specifically later in the crop season (Keeley et al., 1987). PRE herbicides, also referred to as soil residual herbicides, are applied to the soil after crop planting but before emergence for controlling germinating or emerging weed seedlings. Soil-residual PRE herbicides generally lose their residual activity in the soil in 30–50 days; however, most POST herbicides commonly applied in maize have minimal to no soil residual activity (Jhala et al., 2015; Wiggins et al., 2015). Moreover, late-emerging Palmer amaranth plants often escape POST herbicide applications and produce seeds, leading to the replenishment of the soil seedbank and ensuring weed infestations for the next several seasons (Keeley et al., 1987). Therefore, herbicide programs should be focused on season-long Palmer amaranth control to reduce seed production and infestation during subsequent crop seasons. Though over-the-top (broadcast) application of most foliar active POST herbicides is restricted up to certain maize growth stages (Anonymous, 2017a,b,c), some herbicides such as glyphosate and glufosinate can be applied with drop nozzles in the later maize stages extending up to V8–V12 or the 8- to 12-leaf stage and V8–V10 or the 8- to 10-leaf stage in glyphosate- and glufosinate-resistant maize, respectively (Anonymous, 2017b,d). However, the repeated application of herbicides with a single site of action promote the rapid evolution of HR weeds (Délye et al., 2013).

Several soil-residual PRE herbicides have been registered for Palmer amaranth control in maize. For instance, acetochlor, dimethenamid-P, pendimethalin, pyroxasulfone, saflufenacil, or *S*-metolachlor applied PRE provided >80% Palmer amaranth control up to 50 days after application (Johnson et al., 2012; Cahoon et al., 2015; Janak and Grichar, 2016; Meyer et al., 2016). In addition, some soil residual herbicides such as acetochlor, pyroxasulfone, or dimethenamid-P can be applied POST in maize up to certain growth stages (Anonymous, 2017e,f,g). The application of overlapping residual herbicides could be used as an approach for season-long Palmer amaranth control. However, most soil-applied residual herbicides lack foliar activity and are unable to control emerged weeds at the time of application. Therefore, for achieving season-long Palmer amaranth control and to reduce the evolution of HR weeds, different site of action soil-residual herbicides can be applied within 2–3 days of crop planting and in tank-mixture with foliar active herbicides in a POST application.

The cost of herbicide-resistant weed management programs that include different site of action PRE and POST herbicides is usually higher than that of commonly followed weed management practices that involve the use of a single site of action POST herbicide such as glyphosate; therefore, most growers do not consider residual herbicides until they notice the presence of HR weeds in their fields (Peterson, 1999; Norsworthy et al., 2012; Edwards et al., 2014). Additionally, several growers have been avoiding PRE herbicides and relying on POST herbicides to reduce production costs due to low maize and soybean [*Glycine max* (L.) Merr.] commodity prices over the last few years in the USA; however, avoiding PRE herbicides allows early-season crop-weed competition, which could result in a yield penalty (Hall et al., 1992; Schuster and Smeda, 2007). Therefore, it has become crucial to evaluate the economic benefits of implementing herbicide resistant weed management programs to encourage their adoption among growers.

The objectives of this study were to determine the efficacy of soil-residual PRE herbicides fb residual herbicides in tank-mixture with foliar active POST herbicides for PS-II- and HPPD-inhibitor-resistant Palmer amaranth control, crop yield, and net economic return in GR maize. We hypothesized that season-long Palmer amaranth control will be achieved with soil-residual PRE herbicides fb their application in tank-mixture with POST herbicides.

## Materials and methods

### Experimental setup

Field experiments were conducted in 2015 and 2016 in a grower's field confirmed with the presence of PS II- and HPPD-inhibitor-resistant Palmer amaranth near Shickley in Fillmore County, Nebraska (40.46°N, 97.80°E). The level of atrazine resistance was 9- to 14-fold, while the level of resistance to mesotrione, tembotrione, and topramezone was 4-, 4- to 6-, and 14- to 23-fold, respectively, compared to two susceptible Palmer amaranth populations (Jhala et al., 2014). Soil texture at the research site was a Crete silt loam (fine, smectitic, mesic Pachic Udertic Argiustolls) with a pH of 6.5, 26% sand, 57% silt, 17% clay, and 3.5% organic matter. A GR maize hybrid (Mycogen 2D351) was seeded at 87,500 seeds ha^−1^ in rows spaced 76 cm apart on May 30, 2015 and June 1, 2016. The experiment was arranged in a randomized complete block design with four replications and the experimental plots were 3 m wide and 9 m long, consisting of four maize rows. Monthly mean air temperature and total precipitation during the 2015 and 2016 growing seasons and the 30 year average in Shickley, Nebraska is provided in Table 1.

**Table 1:** Monthly mean air temperature and total precipitation during the 2015 and 2016 growing seasons and the 30 year (year) average at Shickley, Nebraska, USA^a^.

**Month**	**Mean temperature**			**Total precipitation**		
	**2015**	**2016**	**30 year average**	**2015**	**2016**	**30 year average**
	^___________^**C**^_____________^			^___________^**mm**^_____________^		
March	7	9	5	12	14	48
April	12	12	11	42	99	68
May	17	16	17	108	200	124
June	23	25	22	264	7	117
July	24	25	25	124	55	86
August	22	23	24	69	147	88
September	22	20	19	104	52	86
October	14	15	12	22	64	59
Annual	12	13	11	908	726	763

a *Mean air temperature and total precipitation data were obtained from the National Weather Service and Cooperative Observer Network (2017)*.

Herbicide programs in the GR maize included PRE-only, POST-only, and PRE fb their sequential application in tank-mixture with POST herbicides, with a total of 15 treatment combinations including a nontreated control (Table 2). The herbicide application timings and rates were based on the label recommendations in maize in Nebraska. Herbicide programs were applied using a CO_2_-pressurized backpack sprayer consisting of a four-nozzle boom fitted with AIXR 110015 flat-fan nozzles (TeeJet Spraying Systems Co., P.O. Box 7900, Wheaton, IL 60189) calibrated to deliver 140 L ha^−1^ at 276 kPa. PRE applications were made within 3 days after planting maize and POST herbicides were applied when Palmer amaranth was 12–15 cm tall.

**Table 2:** Herbicide products, rates, and application timing for control of Photosystem (PS) II- and 4-hydroxyphenylpyruvate dioxygenase (HPPD) inhibitor-resistant Palmer amaranth in glyphosate-resistant maize in field experiments conducted in 2015 and 2016 in Nebraska, USA^a^.

**Herbicide^b^**	**Timing**	**Rate (g ae or ai ha^−1^)**	**Trade name**	**Manufacturer**
Pyroxasulfone	PRE	110	Zidua	BASF Corporation, Research Triangle Park, NC, USA
Topramezone + dimethenamid-P	POST	750	Armezon PRO	BASF Corporation
Pyroxasulfone fb topramezone + dimethenamid-P	PRE fb POST	110 750	Zidua Armezon PRO	BASF Corporation
Saflufenacil + dimethenamid-P	PRE	586	Verdict	BASF Corporation
Saflufenacil + dimethenamid-P fb topramezone + dimethenamid-P	PRE fb POST	586 750	Verdict Armezon PRO	BASF Corporation
Saflufenacil	PRE	75	Sharpen	BASF Corporation
Saflufenacil fb topramezone + dimethenamid-P	PRE fb POST	75 750	Sharpen Armezon PRO	BASF Corporation
Glyphosate	POST	870	Roundup PowerMax	Monsanto Company, 800 North Lindberg Ave., St. Louis, MO, USA
Dicamba + diflufenzopyr	POST	157	Status	BASF Corporation
Pyroxasulfone + saflufenacil fb glyphosate + topramezone + dimethenamid-P + atrazine	PRE fb POST	110 + 75 870 + 750 + 560	Zidua + Sharpen Roundup PowerMax + Armezon PRO + Aatrex	BASF Corporation Monsanto Company + BASF + Syngenta Crop Protection, Inc., Greensboro, NC, USA
Saflufenacil + dimethenamid-P fb glyphosate + topramezone + dimethenamid-P + atrazine	PRE fb POST	586 870 + 750 + 560	Verdict Roundup PowerMax + Armezon PRO + Aatrex	BASF Corporation Monsanto Company + BASF + Syngenta Crop Protection, Inc.
Saflufenacil + dimethenamid-P fb glyphosate + diflufenzopyr + dicamba + pyroxasulfone	PRE fb POST	780 870 + 157 + 91	Verdict Roundup PowerMax + Status + Zidua	BASF Corporation Monsanto Company + BASF + BASF

a *ae, acid equivalent; ai, active ingredient; fb, followed by; PRE, pre-emergence; POST, post-emergence*.

b *All POST herbicide programs were mixed with AMS, ammonium sulfate (DSM Chemicals North America Inc., Augusta, GA) at 2.5% wt/v and NIS, nonionic surfactant (Induce, Helena Chemical Co., Collierville, TN) at 0.25% v/v. No AMS or NIS were added to PRE herbicides. PRE applications were made within 3 d after planting and POST herbicides were applied when Palmer amaranth was 12–15 cm tall*.

Palmer amaranth control was visually estimated at 3 weeks after PRE (WAPRE), before POST herbicide programs were applied, 3 and 6 weeks after POST (WAPOST) herbicide application, and before maize harvest based on a scale of 0–100%, with 0% corresponding to no control and 100% corresponding to plant death. A similar scale was used to assess maize injury at 1 and 2 weeks after PRE and POST herbicide applications, with 0% corresponding to no injury and 100% corresponding to no seed emergence or plant death. Palmer amaranth density was assessed from two randomly selected 0.25 m^2^ quadrats per plot at 3 WAPRE herbicide programs. The Palmer amaranth's aboveground biomass was harvested from two randomly selected 0.25 m^2^ quadrats per plot at 6 WAPOST, oven dried at 65 C for 3 days, and weighed. Palmer amaranth density and biomass data were converted into percent density or biomass reduction compared with the nontreated control (Ganie et al., 2017; Sarangi et al., 2017):

(1) $$\text{Biomass}/\text{Density reduction }(\%)=\frac{(C-B)}{C}\times 100$$

where *C* is the biomass or density of the nontreated control plot and *B* is the biomass or density collected from the experimental plot. At maturity, maize was harvested from the middle two rows of each plot using a plot combine, weighed, and the moisture content were recorded. Maize yields were adjusted to 15.5% moisture content (Ganie et al., 2017).

Economic analysis was performed to evaluate the profit and risk associated with each herbicide program. Net return from herbicide programs was calculated using the maize yield from each replication and herbicide program cost (Bradley et al., 2000; Edwards et al., 2014):

(2) Net return =Gross revenue −Herbicide program cost

Gross revenue was calculated by multiplying the maize yield from each replication for each program by the average grain price ($0.137 kg^−1^) received in Nebraska at harvest time during the experimental years (USDA-NASS, 2016). Each herbicide program cost included the average herbicide cost per hectare obtained from three agricultural chemical dealers in Nebraska and a custom application cost of $18.11 ha^−1^ application^−1^.

### Statistical analysis

Data of Palmer amaranth control estimates, density and aboveground biomass reduction, maize yield, gross return, and net return were subjected to ANOVA using the PROC GLIMMIX procedure in SAS version 9.3 (SAS Institute Inc., Cary, NC 27513). Herbicide programs and experimental years were considered fixed effects, whereas replications were considered a random effect in the model. Data were combined over years when there was no year-by- program interaction. The nontreated control was not included in the data analysis for control estimates and percent density and biomass reduction. Before analysis, data were tested for normality and homogeneity of variance using Shapiro-Wilks goodness-of-fit and Levene's test in SAS. To meet the normality and homogeneity of variance assumptions of ANOVA, all data, except maize yield, were arc-sine square root transformed before analysis; however, back-transformed data are presented with mean separation based on the transformed data. Where the ANOVA indicated herbicide program effects were significant, means were separated at *P* ≤ 0.05 with Tukey-Kramer's pairwise comparison test to reduce type I error for the series of comparisons. Pre-planned single degree-of-freedom contrast analysis was accomplished to compare the relative efficacy of PRE-only, POST-only, and PRE fb POST herbicide programs for Palmer amaranth control, biomass reduction, maize yield, and net return.

## Results

Year-by-herbicide programs interaction was not significant for Palmer amaranth control, density and biomass reduction, maize yield, gross return, and net return; therefore, data were combined over two experimental years.

### Palmer amaranth control

Saflufenacil applied PRE provided 60–69% Palmer amaranth control compared to 36–42% control with pyroxasulfone at 3 WAPRE; however, saflufenacil plus dimethenamid-P premix or pyroxasulfone tank-mixed with saflufenacil provided 76–85% Palmer amaranth control at 3 WAPRE (Table 3). Palmer amaranth control with PRE herbicides applied alone declined to ≤28% later in the season. The contrast analysis suggested that saflufenacil plus dimethenamid-P as well as pyroxasulfone plus saflufenacil provided 80–82% control compared to 65 and 39% control with saflufenacil and pyroxasulfone applied alone, respectively, at 3 WAPRE (Table 4).

**Table 3:** Control of photosystem (PS) II- and 4-hydroxyphenylpyruvate dioxygenase (HPPD)-inhibitor-resistant Palmer amaranth with PRE and/or POST residual herbicides in glyphosate-resistant maize in field experiments conducted in Nebraska, USA in 2015 and 2016^a^.

**Herbicide program^b^**	**Application timing**	**Rate (g ae or ai ha^−1^)**	**Control (%)^c^^,^^d^^,^^e^**			
			**3 WAPRE**	**3 WAPOST**	**6 WAPOST**	**At harvest**
Nontreated Control	–	–	0	0	0	0
Pyroxasulfone	PRE	110	42 de	10 h	5 f	0 f
Topramezone + dimethenamid-P	POST	750	0	57 de	66 b	23 de
Pyroxasulfone fb topramezone + dimethenamid-P	PRE fb POST	110 750	36 e	28 f	39 cd	18 def
Saflufenacil + dimethenamid-P	PRE	586	78 abc	19 gf	26 de	28 de
Saflufenacil + dimethenamid-P fb topramezone + dimethenamid-P	PRE fb POST	586 750	80 abc	64 d	73 b	65 bc
Saflufenacil	PRE	75	60 cd	8 gh	15 ef	12 ef
Saflufenacil fb topramezone + dimethenamid-P	PRE fb POST	75 750	69 bc	36 ef	58 bc	40 cd
Glyphosate	POST	870	0	86 bc	95 a	88 ab
Dicamba + diflufenzopyr	POST	157	0	69 cd	91 a	89 a
Pyroxasulfone + saflufenacil fb glyphosate + topramezone + dimethenamid-P + atrazine	PRE fb POST	110 + 75 870 + 750 + 560	85 a	98 a	99 a	99 a
Saflufenacil + dimethenamid-P fb glyphosate + topramezone + dimethenamid-P + atrazine	PRE fb POST	586 870 + 750 + 560	76 abc	95 a	99 a	99 a
Saflufenacil + dimethenamid-P fb glyphosate + diflufenzopyr + dicamba + pyroxasulfone	PRE fb POST	780 870 + 157 + 91	83 ab	98 a	99 a	99 a
Saflufenacil + dimethenamid-P fb glyphosate + diflufenzopyr + pendimethalin	PRE fb POST	780 870 + 157 + 1,060	83 ab	98 a	98 a	98 a
Pyroxasulfone + saflufenacil fb glyphosate + diflufenzopyr + dicamba + atrazine	PRE fb POST	110 + 75 870 + 157 + 1,120	79 ab	98 a	99 a	99 a
S.E			4.6	5.4	5.2	6.0
**CONTRASTS^f^**						
PRE vs. POST	–	–	–	12 vs. 71^*^	15 vs. 84^*^	13 vs. 66^*^
PRE vs. PRE fb POST	–	–	–	12 vs. 77^*^	15 vs. 83^*^	13 vs. 77^*^
POST vs. PRE fb POST	–	–	–	71 vs. 77^*^	84 vs. 83^**^	66 vs. 77^*^

a *ae, acid equivalent; ai, active ingredient; fb, followed by; PRE, pre-emergence; POST, post-emergence; S.E, standard error*.

b *All POST herbicide program were mixed with AMS, ammonium sulfate (DSM Chemicals North America Inc., Augusta, GA) at 2.5% wt/v and NIS, nonionic surfactant (Induce, Helena Chemical Co., Collierville, TN) at 0.25% v/v. No AMS or NIS were added to PRE herbicides. PRE applications were made within 3 d after planting and POST herbicides were applied when Palmer amaranth was 12–15 cm tall*.

c *Year-by- program interaction for Palmer amaranth control was not significant; therefore, data were combined over 2 years. Data were arc-sine square-root transformed before analysis; however, data presented are the means of actual values for comparison based on interpretation from the transformed values*.

d *The nontreated control data was not included in the statistical analysis*.

e *Means within columns with no common letter(s) are significantly different according to Tukey–Kramer's pairwise comparison test at P ≤ 0.05*.

f Single degree-of-freedom contrast analysis;

* *significant (p < 0.05)*;

** *non-significant (p > 0.05)*.

**Table 4:** Contrast means for control and density reduction of photosystem (PS) II- and 4-hydroxyphenylpyruvate dioxygenase (HPPD) inhibitor-resistant Palmer amaranth at 3 weeks after pre-emergence herbicide application in glyphosate-resistant maize in field experiments conducted in Nebraska, USA in 2015 and 2016^a^.

**Herbicide program**	**Control (%)**	**Density reduction^b^ (%)**
Pyroxasulfone vs. Saflufenacil	39 vs. 65^*^	28 vs. 58^*^
Pyroxasulfone + Saflufenacil vs. Saflufenacil + Dimethenamid-P	82 vs. 80^**^	78 vs. 66^**^
Saflufenacil vs. Pyroxasulfone + Saflufenacil	65 vs. 82^*^	58 vs. 78^*^

a Single degree-of-freedom contrast analysis;

* significant (p < 0.05);

** *non-significant (p > 0.05)*.

b *Palmer amaranth density data were converted into percent density reduction compared with the nontreated control using the formula: $Densityreduction\;\left(\%\right)=\frac{\left(C-B\right)}{C}\times \text{100}$, where C is the density of the nontreated control plot and B is the density collected from the experimental plot*.

**Figure 1:**
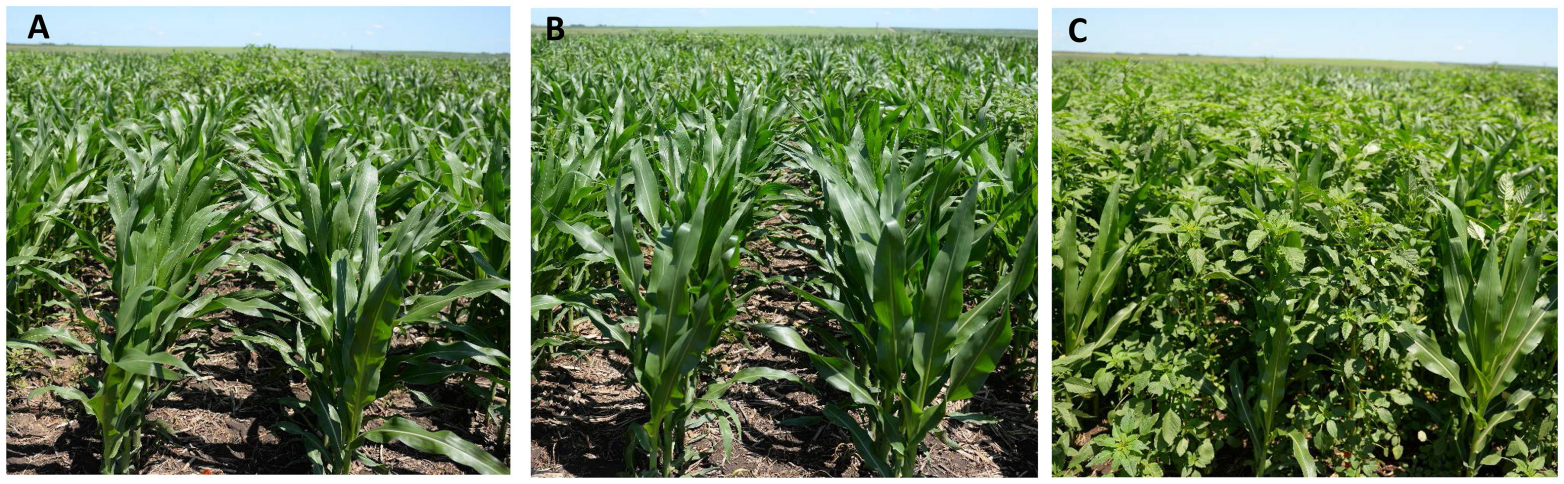
Control of Photosystem (PS) II- and 4-hydroxyphenylpyruvate dioxygenase (HPPD) inhibitor-resistant Palmer amaranth with **(A)** pyroxasulfone + saflufenacil followed by (fb) glyphosate + topramezone + dimethenamid-P + atrazine, and **(B)** saflufenacil + dimethenamid-P fb glyphosate + diflufenzopyr + pendimethalin compared to **(C)** nontreated control at 3 weeks after post-emergence.

At 3 and 6 WAPOST, Palmer amaranth control ranged from 86 to 95% with glyphosate compared to 57–66% and 69–95% control with topramezone plus dimethenamid-P and diflufenzopyr plus dicamba, respectively. Palmer amaranth control was reduced to 23% at harvest with a POST-only application of topramezone plus dimethenamid-P compared to glyphosate (88%) and dicamba plus diflufenzopyr (89%). However, ≥95% Palmer amaranth control was achieved with pyroxasulfone plus safluefenacil, or saflufenacil plus dimethenamid-P applied PRE fb glyphosate plus topramezone plus dimethenamid-P plus atrazine, glyphosate plus diflufenzopyr plus dicamba plus pyroxasulfone, glyphosate plus diflufenzopyr plus pendimethalin, or glyphosate plus diflufenzopyr plus dicamba plus atrazine at 3 and 6 WAPOST, and at harvest (Figure 1; Table 3). Most PRE fb POST herbicide programs resulted in ≥95% Palmer amaranth control throughout the season, except pyroxasulfone fb topramezone plus dimethenamid-P (18–39%), saflufenacil fb topramezone plus dimethenamid-P (36–58%), or saflufenacil plus dimethenamid-P fb topramezone plus dimethenamid-P (64–73%) (Table 3). The contrast analysis indicated that PRE fb POST programs provided greatest Palmer amaranth control (77%) compared to POST-only programs (66–71%) at 3 WAPOST and at harvest; however, similar control (83–84%) was achieved at 6 WAPOST (Table 3). Similarly, POST-only programs provided 66–71% control compared to <15% control with PRE-only programs at 3 and 6 WAPOST and at harvest.

### Palmer amaranth density and biomass reduction

Palmer amaranth density was reduced by 55–82% with saflufenacil, pyroxasulfone plus saflufenacil, or saflufenacil plus dimethenamid-P compared to 27–29% density reduction with pyroxasulfone at 3 WAPRE. Based on the contrast analysis, pyroxasulfone plus saflufenacil or saflufenacil plus dimethenamid-P provided the greatest density reduction (66–78%) compared to saflufenacil (58%) and pyroxasulfone (39%) (Table 4).

Saflufenacil or saflufenacil plus dimethenamid-P applied PRE alone provided 29–56% biomass reduction compared to no biomass reduction with pyroxasulfone at 6 WAPOST (Table 5). Glyphosate or dicamba plus diflufenzopyr applied POST alone resulted in ≥97% biomass reduction compared to 66% biomass reduction with topramezone plus dimethenamid-P. The PRE fb POST programs provided 76–99% Palmer amaranth biomass reduction (Figure 1), except for saflufenacil fb topramezone plus dimethenamid-P (69%), and pyroxasulfone fb topramezone plus dimethenamid-P (44%) at 6 WAPOST (Table 5). The contrast analysis indicated that PRE fb POST programs provided 99% Palmer amaranth biomass reduction compared to POST-only (87%) and PRE-only programs (28%) at 6 WAPOST (Table 5).

**Table 5:** Effect of herbicide programs on photosystem (PS) II- and 4-hydroxyphenylpyruvate dioxygenase (HPPD)-inhibitor-resistant Palmer amaranth density reduction at 3 weeks after PRE, biomass reduction at 6 weeks after POST, maize injury at 2 weeks after PRE, and maize yield at harvest in glyphosate-resistant maize in field experiments conducted in Nebraska, USA in 2015 and 2016^a^.

**Herbicide program^b^**	**Application timing**	**Rate (g ae or ai ha^−1^)**	**Density reduction^c^^,^^d^^,^^e^^,^^f^**	**Biomass reduction^c^^,^^d^^,^^e^^,^^f^**	**Maize yield^e, f^ (kg ha^−1^)**
			**3 WAPRE (%)**	**6 WAPOST (%)**	
Nontreated Control	–	–	0	0	1,042 f
Pyroxasulfone	PRE	110	27 bc	0	1,870 ef
Topramezone + dimethenamid-P	POST	750	0	66 bcde	8,525 cd
Pyroxasulfone fb topramezone + dimethenamid-P	PRE fb POST	110 750	29 bc	44 def	5,600 de
Saflufenacil + dimethenamid-P	PRE	586	67 ab	56 bcde	4,108 ef
Saflufenacil + dimethenamid-P fb topramezone + dimethenamid-P	PRE fb POST	586 750	88 a	76 abcd	11,450 bc
Saflufenacil	PRE	75	55 ab	29 ef	1,990 ef
Saflufenacil fb topramezone + dimethenamid-P	PRE fb POST	75 750	60 ab	69 cde	9,194 cd
Glyphosate	POST	870	0	98 a	14,324 ab
Dicamba + diflufenzopyr	POST	157	0	97 a	11,440 bc
Pyroxasulfone + saflufenacil fb glyphosate + topramezone + dimethenamid-P + atrazine	PRE fb POST	110 + 75 870 + 750 + 560	74 a	99 a	16,044 a
Saflufenacil + dimethenamid-P fb glyphosate + topramezone + dimethenamid-P + atrazine	PRE fb POST	586 870 + 750 + 560	49 ab	99 a	17,161 a
Saflufenacil + dimethenamid-P fb glyphosate + diflufenzopyr + dicamba + pyroxasulfone	PRE fb POST	780 870 + 157 + 91	59 ab	99 a	17,114 a
Saflufenacil + dimethenamid-P fb glyphosate + diflufenzopyr + pendimethalin	PRE fb POST	780 870 + 157 + 1,060	64 ab	99 a	16,031 a
Pyroxasulfone + saflufenacil fb glyphosate + diflufenzopyr + dicamba + atrazine	PRE fb POST	110 + 75 870 + 157 + 1,120	82 a	99 a	16,346 a
S.E			19	11	1,082
**CONTRASTS^g^**					
PRE vs. POST	–	–	–	28 vs. 87^*^	2,656 vs. 11,429^*^
PRE vs. PRE fb POST	–	–	–	28 vs. 99^*^	2,656 vs. 13,617^*^
POST vs. PRE fb POST	–	–	–	87 vs. 99^*^	11,429 vs. 13,617^*^

a *ae, acid equivalent; ai, active ingredient; fb, followed by; PRE, pre-emergence; POST, post-emergence; S.E, standard error*.

b *All POST herbicide programs were mixed with AMS, ammonium sulfate (DSM Chemicals North America Inc., Augusta, GA) at 2.5% wt/v and NIS, nonionic surfactant (Induce, Helena Chemical Co., Collierville, TN) at 0.25% v/v. No AMS or NIS were added to PRE herbicides. PRE applications were made within 3 d after planting and POST herbicides were applied when Palmer amaranth was 12–15 cm tall*.

c *Data were arc-sine square-root transformed before analysis; however, data presented are the means of actual values for comparison based on interpretation from the transformed values*.

d *Percent density and biomass reduction data of non-treated control were not included in analysis. Palmer amaranth density and biomass data were converted into percent density or biomass reduction compared with the nontreated control plots using the formula: $Biomass/Densityreduction\;\left(\%\right)=\frac{\left(C-B\right)}{C}\times \text{100}$, where C is the biomass or density of the nontreated control plot and B is the biomass or density collected from the experimental plot*.

e *Year-by-program interaction was not significant; therefore, data were combined over 2 experimental years*.

f *Means within columns with no common letter(s) are significantly different according to Tukey–Kramer's pairwise comparison test at P ≤ 0.05*.

g Single degree-of-freedom contrast analysis;

* *significant (p < 0.05)*.

### Maize injury and yield

Herbicide injury on maize was negligible (0–6%) and transient without impact on maize yield (data not shown). The nontreated control resulted in the lowest maize yield of 1,042 kg ha^−1^ and was comparable with PRE-only programs including pyroxasulfone (1,870 kg ha^−1^), saflufenacil (1,990 kg ha^−1^), or saflufenacil plus dimethenamid-P (4,108 kg ha^−1^). Most of the PRE fb POST programs resulted in greater maize yield varying from 16,031 to 17,161 kg ha^−1^, except for pyroxasulfone fb topramezone plus dimethenamid-P (5,600 kg ha^−1^), saflufenacil fb topramezone plus dimethenamid-P (9,194 kg ha^−1^), and saflufenacil plus dimethenamid-P fb topramezone plus dimethenamid-P (11,450 kg ha^−1^) (Table 5). Maize yield with POST-only programs varied from 8,525 to 14,324 kg ha^−1^ and glyphosate applied alone resulted in a yield comparable with the highest yielding PRE fb POST programs. The contrast analysis indicated that PRE fb POST programs provided higher (13,617 kg ha^−1^) maize yield compared to POST-only (11,429 kg ha^−1^) and PRE-only (2,656 kg ha^−1^) programs (Table 5).

### Economic analysis

The cost of PRE-only and POST-only herbicide programs varied from US $61.01 to US $98.11 ha^−1^ and US $29.79 to US $65.90 ha^−1^, respectively, compared with $133.00 to $215.64 ha^−1^ for PRE fb POST programs (Table 6). The gross income and net returns were in consensus with the yield (Tables 5, 6). The PRE fb POST herbicide programs including pyroxasulfone plus safluefenacil, or saflufenacil plus dimethenamid-P applied PRE fb glyphosate plus topramezone plus dimethenamid-P plus atrazine, glyphosate plus diflufenzopyr plus dicamba plus pyroxasulfone, glyphosate plus diflufenzopyr plus pendimethalin, or glyphosate plus diflufenzopyr plus dicamba plus atrazine applied POST provided the highest net returns ranging from $2,023 to $2,246 ha^−1^ (Table 6). The net returns with PRE-only programs were < $475 ha^−1^ compared to $1,123 to $1,965 ha^−1^ with POST-only herbicide programs, signifying the importance of POST programs (Table 6). The contrast analysis suggested that PRE fb POST programs provided the highest ($1,724 ha^−1^) net return fb POST-only ($1,539 ha^−1^), and PRE-only ($285 ha^−1^) programs.

**Table 6:** Cost of herbicide programs for controlling photosystem (PS) II- and 4-hydroxyphenylpyruvate dioxygenase (HPPD) inhibitor-resistant Palmer amaranth and net income from maize yield in glyphosate-resistant maize in field experiments conducted in Nebraska, USA in 2015 and 2016^a^.

**Herbicide program^b^**	**Application timing**	**Rate (g ae or ai ha^−1^)**	**Program cost^c^ ($ ha^−1^)**	**Gross income^d^ ($ ha^−1^)**	**Net return^e^^,^^f^^,^^g^ ($ ha^−1^)**
Nontreated Control	–	–	0	145 f	145 f
Pyroxasulfone	PRE	110	91.76	260.76 ef	169 f
Topramezone + dimethenamid-P	POST	750	65	1,188 cd	1,123 de
Pyroxasulfone fb topramezone + dimethenamid-P	PRE fb POST	110 750	156.76	779.76 de	623 ef
Saflufenacil + dimethenamid-P	PRE	586	98.11	572.11 ef	474 f
Saflufenacil + dimethenamid-P fb topramezone + dimethenamid-P	PRE fb POST	586 750	133.11	1,595 bc	1,462 cd
Saflufenacil	PRE	75	61.01	274 ef	213 f
Saflufenacil fb topramezone + dimethenamid-P	PRE fb POST	75 750	136.01	1,281 cd	1,145 de
Glyphosate	POST	870	29.79	1,995 ab	1,965 abc
Dicamba + diflufenzopyr	POST	157	65.9	1,594 bc	1,528 bcd
Pyroxasulfone + saflufenacil fb glyphosate + topramezone + dimethenamid-P + atrazine	PRE fb POST	110 + 75 870 + 750 + 560	211.18	2,234 a	2,023 ab
Saflufenacil + dimethenamid-P fb glyphosate + topramezone + dimethenamid-P + atrazine	PRE fb POST	586 870 + 750 + 560	144.23	2,390 a	2,246 a
Saflufenacil + dimethenamid-P fb glyphosate + diflufenzopyr + dicamba + pyroxasulfone	PRE fb POST	780 870 + 157 + 91	215.64	2,384 a	2,168 a
Saflufenacil + dimethenamid-P fb glyphosate + diflufenzopyr + pendimethalin	PRE fb POST	780 870 + 157 + 1,060	172.51	2,233 a	2,060 a
Pyroxasulfone + saflufenacil fb glyphosate + diflufenzopyr + dicamba + atrazine	PRE fb POST	110 + 75 870 + 157 + 1,120	212.04	2,276 a	2,064 a
S.E.			–	150	150
**CONTRASTS^h^**					
PRE vs. POST	–	–	–	–	285 vs. 1,539^*^
PRE vs. PRE fb POST	–	–	–	–	285 vs. 1,724^*^
POST vs. PRE fb POST	–	–	–	–	1,539 vs. 1,724^*^

a *ae, acid equivalent; ai, active ingredient; fb, followed by; PRE, pre-emergence; POST, post-emergence; S.E, standard error*.

b *All POST herbicide programs were mixed with AMS, ammonium sulfate (DSM Chemicals North America Inc., Augusta, GA) at 2.5% wt/v and NIS, nonionic surfactant (Induce, Helena Chemical Co., Collierville, TN) at 0.25% v/v. No AMS or NIS were added to PRE herbicides. PRE applications were made within 3 d after maize planting and POST herbicides were applied when Palmer amaranth was 12–15 cm tall*.

c *Program cost includes the average cost of herbicide, AMS, and NIS; and the cost of application (US $18.11 ha^−1^ application^−1^) from two independent sources in Nebraska*.

d *Gross revenue was calculated by multiplying maize yield for each program by the average grain price received in Nebraska at harvest time during the experimental years ($0.137 kg^−1^, USDA-NASS, 2016)*.

e *Net return was calculated as gross income from glyphosate-resistant maize yield minus herbicide program cost*.

f *Data were arc-sine square-root transformed before analysis; however, data presented are the means of actual values for comparison based on interpretation from the transformed values. Year-by- program interaction was not significant; therefore, data were combined over two experimental years*.

g *Means within columns with no common letter(s) are significantly different according to Tukey-Kramer's pairwise comparison test P ≤ 0.05*.

h Single degree-of-freedom contrast analysis;

* significant (p < 0.05);

^**^non-significant (p > 0.05).

## Discussion

The results indicated that PRE programs with multiple sites of action, including saflufenacil plus dimethenamid-P premix or pyroxasulfone tank-mixed with saflufenacil provided higher control (80–82%) compared to saflufenacil or pyroxasulfone applied alone (39–65%) at 3 WAPRE. Similarly, Kohrt and Sprague (2017) reported 75% Palmer amaranth control with saflufenacil applied alone and 80–97% control when saflufenacil was tank-mixed with pyroxasulfone at 45 DAPRE in a 3-year field study in Michigan. Janak and Grichar (2016) also reported >95% Palmer amaranth control with saflufenacil plus dimethenamid-P at 95 DAPRE in maize production fields in Texas. Similarly, Aulakh and Jhala (2015) reported 96% common waterhemp control with saflufenacil plus dimethenamid-P at 15 DAPRE in soybean in Nebraska.

The POST herbicide programs including tank-mixture of active ingredients with residual activity and multiple sites of action provided 95–99% Palmer amaranth control compared to topramezone plus dimethenamid-P (57–70%) (Figure 1; Table 3). Similarly, Wiggins et al. (2015) reported 95–99% control of GR Palmer amaranth with glyphosate plus *S*-metolachlor plus mesotrione plus atrazine, thiencarbazone-methyl plus tembotrione plus atrazine, or glyphosate plus atrazine at 28 DAPOST. However, the unacceptable control with topramezone plus dimethenamid-P may be attributed to a high-level resistance (14- to 23-fold) of Palmer amaranth to topramezone (Jhala et al., 2014). In the same study, Jhala et al. (2014) reported only 58% control of PS II- and HPPD-inhibitor-resistant Palmer amaranth with topramezone compared to 99% control of the susceptible biotypes or 87–99% control of resistant Palmer amaranth when topramezone was tank-mixed with atrazine at 21 DAPOST. Although Palmer amaranth was resistant to PRE and POST applied atrazine or topramezone, the POST application of glyphosate plus topramezone plus dimethenamid-P plus atrazine controlled Palmer amaranth ≥95% throughout the season due to the synergistic interactions of atrazine and topramezone as well as the presence of glyphosate (Table 3). Previous studies have reported synergistic interaction when a PS-II inhibitor such as atrazine is applied in tank-mixture with an HPPD inhibitor for POST weed control in maize (Abendroth et al., 2006; Hugie et al., 2008), including control of atrazine- and HPPD-inhibitor-resistant Palmer amaranth (Jhala et al., 2014). Similarly, synergistic interaction between HPPD- and PS II-inhibiting herbicides has been reported for control of giant ragweed (*Ambrosia trifida* L.), common lambsquarters (*Chenopodium album* L.), velvetleaf (*Abutilon theophrasti* Medik.), common waterhemp, and redroot pigweed (*Amaranthus retroflexus* L.) (Abendroth et al., 2006; Hugie et al., 2008; Woodyard et al., 2009a,b). At the physiological level, atrazine binds at the Q_B_ binding site of DI protein of PS II and inhibits the electron transport during photosynthesis (Fuerst and Normanm, 1991). On the other hand, mesotrione inhibits HPPD enzyme synthesis which leads to depletion of plastoquinone resulting in decreasing electron transport during photosynthesis and also inhibit carotenoids and tocopherols synthesis (Hess, 2000; Mitchell et al., 2001; McCurdy et al., 2008). Both PS II- and HPPD-inhibitors block the electron transport in PS II due to their complementary mode of action and lead to the accumulation of reactive oxygen species and free radicals that damage the foliar tissue membranes (Hess, 2000).

The POST-only programs including glyphosate or dicamba plus diflufenzopyr resulted in 88–95% Palmer amaranth control and were comparable with the PRE fb POST herbicide programs, except at 3 WAPOST (Table 3). Jhala et al. (2014) reported 90–99% control of the same Palmer amaranth biotype with glyphosate, glufosinate, or dicamba at 21 DAPOST in a greenhouse study. Similarly, Norsworthy (2004) reported 100% Palmer amaranth control with a single or sequential application of glyphosate at 5 WAPOST. Likewise, Crow et al. (2016) reported >87% control of >20 cm tall Palmer amaranth with dicamba plus diflufenzopyr applied alone or in tank-mixture with glyphosate, mesotrione, tembotrione, mesotrione plus rimsulfuron, or tembotrione plus thiencarbazone at 4 WAPOST. Nonetheless, the dependence on POST herbicides with a single site of action must be avoided to prevent the evolution of HR weeds (Chahal and Jhala, 2015; Chahal et al., 2017; Ganie and Jhala, 2017b). Furthermore, the confirmation of GR Palmer amaranth in a GR soybean/maize production field in south-central Nebraska signifies that dependence on a single POST herbicide program is not a reliable option (Chahal et al., 2017).

Weed density at the time of POST herbicide application plays an important role in determining herbicide efficacy and the number of weeds surviving (Dieleman et al., 1999). Bell et al. (2015) reported that flumioxazin plus pyroxasulfone applied PRE in soybean reduced Palmer amaranth emergence and demonstrated a potential to enhance the efficacy of POST herbicides and reduce selection pressure by exposing a lower number of Palmer amaranth plants to POST herbicides. Similarly, Meyer et al. (2016) reported ≥97% control of Palmer amaranth and common waterhemp for more than 3 weeks of applying isoxaflutole plus *S*-metolachlor plus metribuzin, *S*-metolachlor plus mesotrione, or flumioxazin plus pyroxasulfone. In addition, the application of residual herbicides in a tank-mixture with a foliar active POST herbicide is obligatory for the season-long control of Palmer amaranth because of its extended emergence period that typically begins from early May to late September (Jha and Norsworthy, 2009; Ward et al., 2013).

Palmer amaranth density and biomass reduction were in consensus with the visual estimates of Palmer amaranth control at 3 WAPRE and 6 WAPOST, respectively. Jhala et al. (2014) and Kohrt and Sprague (2017) reported an agreement between Palmer amaranth control estimates and biomass reduction with herbicide programs tested. The PRE fb POST programs resulted in greater yield compared to a PRE- or POST-only herbicide program, excluding a POST-only application of glyphosate. Although higher rainfall was received at the experimental site in 2015 during the critical period of maize growth from the V2 to V8 development stages during June and July compared to 2016 and the 30-year average (Table 1), no difference in Palmer amaranth control, density and biomass reduction, and maize yield was observed between the two experimental years. However, previous studies have reported greater weed control and maize yield in years receiving higher rainfall compared to dry years (Whaley et al., 2009; Petcu et al., 2015). A higher level of Palmer amaranth control in this study with glyphosate was due to the fact that glyphosate had not been applied at the research site for the last 8 years because the field was under continuous maize seed production (Jhala et al., 2014). Similarly, the economic analysis indicated higher net returns with PRE fb POST herbicide programs with multiple herbicide sites of action even though the diversified herbicide mixtures were more expensive. Bradley et al. (2000) also reported that PRE fb POST programs including acetochlor or *S*-metolachlor applied PRE fb dicamba or glufosinate plus atrazine applied POST were among the high net income-producing programs with excellent weed control in maize. Norsworthy (2004) also reported greater gross profit with chlorimuron plus metribuzin or sulfentrazone applied PRE fb glyphosate compared to glyphosate without PRE herbicide applications.

## Conclusion

The evolution of PS II- and HPPD-inhibitor-resistant Palmer amaranth has become a concern for field maize, maize grown for popcorn and seed production in Nebraska, USA. The results of this study suggested that season-long Palmer amaranth management is possible by including overlapping residual herbicides in synergistic tank-mixtures of PS II- and HPPD-inhibiting herbicides. In addition, application of PS II- and HPPD-inhibiting herbicides in a tank-mixture with glyphosate, dicamba plus dimethenamid-P, or pyroxasulfone provided an effective strategy for Palmer amaranth control due to the synergistic action of atrazine and topramezone; along with the residual activity of atrazine, dimethenamid-P, or pyroxasulfone; and glyphosate or dicamba as additional effective sites of action to reduce selection pressure. However, Culpepper (2006) emphasized that no single herbicide program will provide a consistently satisfactory control of Palmer amaranth for more than a 4- to 5-year period. Therefore, it has become crucial to incorporate feasible non-chemical weed control tools including tillage, rotation of different HR cultivars with conventional crop cultivars, row spacing, and harvest weed seed control etc., for an integrated HR Palmer amaranth management.

## Author contributions

PC conducted the experiments, analyzed the data, and edited the manuscript; ZG assisted in the experiments and writing of the manuscript; and AJ conceptualized and designed the research and edited the manuscript.

### Conflict of interest statement

The authors declare that the research was conducted in the absence of any commercial or financial relationships that could be construed as a potential conflict of interest.

## Abbreviations

fb: followed by
PRE: pre-emergence
POST: post-emergence
WAPRE: weeks after pre-emergence
WAPOST: weeks after post-emergence.
